# Fuzzy Clustering Algorithm Based on Improved Global Best-Guided Artificial Bee Colony with New Search Probability Model for Image Segmentation

**DOI:** 10.3390/s22228956

**Published:** 2022-11-18

**Authors:** Waleed Alomoush, Osama A. Khashan, Ayat Alrosan, Essam H. Houssein, Hani Attar, Mohammed Alweshah, Fuad Alhosban

**Affiliations:** 1 School of Information Technology, Skyline University College, Sharjah P.O. Box 1797, United Arab Emirates; 2 Research and Innovation Centers, Rabdan Academy, Abu Dhabi P.O. Box 114646, United Arab Emirates; 3 Faculty of Computers and Information, Minia University, Minia 61519, Egypt; 4 Department of Energy Engineering, Zarqa University, Zarqa 13132, Jordan; 5 Prince Abdullah Bin Ghazi Faculty of Information and Communication Technology, Al-Balqa Applied University, Al-Salt 19117, Jordan; 6 CIS Department, Faculty of Computer Information Systems, Higher Colleges of Technology, Dubai P.O. Box 16062, United Arab Emirates

**Keywords:** data clustering, artificial bee colony, centroids location, natural images and validity index

## Abstract

Clustering using fuzzy C-means (FCM) is a soft segmentation method that has been extensively investigated and successfully implemented in image segmentation. FCM is useful in various aspects, such as the segmentation of grayscale images. However, FCM has some limitations in terms of its selection of the initial cluster center. It can be easily trapped into local optima and is sensitive to noise, which is considered the most challenging issue in the FCM clustering algorithm. This paper proposes an approach to solve FCM problems in two phases. Firstly, to improve the balance between the exploration and exploitation of improved global best-guided artificial bee colony algorithm (IABC). This is achieved using a new search probability model called PIABC that improves the exploration process by choosing the best source of food which directly affects the exploitation process in IABC. Secondly, the fuzzy clustering algorithm based on PIABC, abbreviated as PIABC-FCM, uses the balancing of PIABC to avoid getting stuck into local optima while searching for the best solution having a set of cluster center locations of FCM. The proposed method was evaluated using grayscale images. The performance of the proposed approach shows promising outcomes when compared with other related works.

## 1. Introduction

The main goal of image segmentation methods is to divide an image into many regions that are made of groups of pixels. Image segmentation is utilized to set elements within an image and the result of this process is a combination of regions that cover the entire pixels within the image. Objects in the same segment have identical characteristics, and hence, the pixels have a similar texture, color, and intensity. These characteristics are significantly different between adjacent regions [1,2]. In recent years, combined processes have been used in image segmentation methods such as evolutionary algorithms, data clustering, and artificial neural networks, and these processes have been gaining much attention [3,4,5,6].

Many limitations in real image applications, which include issues of region boundaries overlapping and intensity inhomogeneities, poor contrast, and spatial resolution lead to a complex process of image segmentation [7]. To address this big obstacle, the theory of fuzzy set was introduced to allow partial membership categorization using a membership fitness equation. A soft segmentation method-based fuzzy clustering algorithm has been widely studied and successfully applied in image segmentation [8]. Among the fuzzy clustering methods, the FCM [9] algorithm is a popular method that is used in image segmentation for its robust characteristics for ambiguity and because it can retain much more information than the hard segmentation methods [7,10]

In this regard, fuzzy-clustering-based segmentation approaches have numerous advantages as the boundaries between segments are unclear in most images and the majority of the images show uncertain regional boundaries. Fuzzy clustering is considered to be extremely promising because it can cope with these kinds of data characteristics. Therefore, the FCM algorithm is considered the most extensively utilized algorithm [7,11,12,13,14]. Clustering methods and segmentation of images have a similar aim which is to determine the appropriate classification. Based on some characteristics, the clustering approaches are assigned a pixel in each region [3,7]

Conventional FCM algorithm, however, suffers from some drawbacks such as being sensitive to noise as it is only useful on noise-free images. Accordingly, a critical limitation of the FCM algorithm is that it does not provide any details concerning the spatial context, which leads to being very sensitive to noise and imaging artifacts [15]. The objects (pixels) on a grey image are rather overlapping, i.e., the adjacent pixels have nearly the same characteristics. Moreover, the selection of the initial cluster’s center, stuck into local optimum and sensitive to noise, are among the most common weaknesses of the FCM clustering algorithm.

Metaheuristic search algorithms such as firefly algorithm (FA) [16,17], ant colony optimization (ACO) [18], cuckoo search (CS) [19,20], particle swarm optimization (PSO) [21], harmony search (HS) [22,23,24], and artificial bee colony, namely, (ABC) [25], are conveniently used in many fields including in segmentation of images [1,26,27,28,29,30], clustering [31], and also used in several domains [32,33,34]. The clustering-approach-based metaheuristic search algorithms have been classified as a superior method for such a problem.

This paper proposed an approach to solve FCM problems over two phases. In the first phase, a new search probability model is proposed based on improved global best-guided IABC, which is called PIABC. This new search probability model, PIABC, can improve the exploration process that directly affects the exploitation process in IABC. In the second phase, a fuzzy clustering algorithm based on PIABC abbreviated as PIABC-FCM uses the balancing of PIABC to prevent becoming trapped into local optima while searching for the best solution as location centroids of FCM. The content of this article is organized as follows: fuzzy c-means (FCM) is introduced in part two, the original ABC is introduced in part three, the literature review and related work are shown in part four, and the proposed methods are introduced in part five. Finally, in part six, the experiments and results are discussed, while the conclusion is displayed in part seven.

## 2. Related Work

Several initiatives about segmentation methods of clustering-based FCM were touched on in recent years. Therefore, clustering-based metaheuristic search algorithms are considered a suitable choice for such a problem. In this section, many related studies are reviewed as follows.

The ABC algorithm and the standard of FCM have been coupled in an image segmentation method, namely, FABC, which was proposed in Bose and Mali [7]. The fuzzy membership fitness value has been employed by FABC to locate the best cluster centers using ABC. In this study, the researchers used FABC, GA, PSO, and EM on a variety of greyscale images, including synthetic, medical, and textural images. When compared with certain other relevant studies, FABC is more efficient for both validity indices and accuracy measurements.

Furthermore, Ref. [35] developed a new method-based FCM and Modified ABC algorithm, namely, MoAB-FCM. The results revealed that the MoABC-FCM approach improved the basic FCM’s performance and outperforms other related studies that used optimization search techniques. The work in [36] presented a new version of fuzzy clustering based on the ABC algorithm, namely, ABC-SFCM. This approach has been carried out using two kinds of images such as synthetic and real images. The authors in [5] presented a new version of ABC-SFCM based on MeanABC and reformulated objective function, namely, MeanABC-SFCM. Furthermore, Hancer, et al. [37] presented an image segmentation method using ABC to determine brain tumors from the MRI brain image. In [30], fuzzy c-means and ABC algorithms were coupled, which is called ABC-FCM, and the new approach has been tested using two types of MRI images, namely, the simulated brain data and actual MRI images. The work in [29] proposed a new kind of ABC algorithm, called MeanABC, which is a hybrid with the FCM algorithm. This algorithm has been applied to IBSR brain MRI images.

Meanwhile, Yu, et al. [38] proposed a new ACO optimization-based FCM clustering algorithm and it was obtained on image segmentation. In this work, FCM memberships of objective function were improved based on spatial information which improved the algorithm’s accuracy and performance, which led to enhancing the algorithm’s precision and effectiveness of the image segmentation process. The experiments were chosen to test the quality of the proposed algorithm, and the accuracy of the ACO-FCM outcome was indicated to become an established image segmentation method.

An automatic image segmentation algorithm, namely, DCPSO, was presented in [39,40,41]. In this proposed algorithm, the number of clusters were chosen based on the binary PSO process, which is used to find the centroid location of fuzzy K-means. This approach has been carried out using two kinds of images such as natural and synthetic images. Further, [42] introduced a new segmentation method using PSO based on the outlier rejection and level set. The results of this method were compared with related works, and it shows more effectiveness.

In [43], the authors used harmony search (HS) to determine the location of the centroid for FCM, namely, HS-FCM. The proposed algorithm experimented on the data of MRI images and the outcomes were promising. Additionally, [14] introduced an automatic image segmentation method using hybrid HS with FCM which is called DCHS. The proposed method was carried out on MRI brain images, both real and simulated. Based on the results, the DCHS approach can determine the suitable number of segments in brain MRI images.

Further, [26] presented a segmentation method, namely, FFCM. They used a hybrid method of firefly algorithm (FA) and FCM. This approach was carried out on simulated and real brain datasets. The analysis of the results were encouraging when compared with related works. However, FFCM still has a sensitivity to noise problems, which is the main weakness of FCM [28]. Moreover, the authors in [4] presented an automatic segmentation-method-based hybrid firefly mate list with FCM, namely, (AUTO-FCM-FMA). The proposed algorithm has been applied to grayscale images, and the experiments and outcomes were encouraging when compared with related works. Meanwhile, [3] proposed biogeography-based optimization (BBO) and the combination of FCM with a group of enhanced BBO algorithms. The experiment was performed on a group of natural images.

## 3. FCM

One of the unsupervised classification approaches is FCM, which can divide groups of objects based on the similarity level, with the main goal of increasing the similarity of objects within a cluster while decreasing the similarity among objects between different clusters [44,45]. However, FCM is sensitive to noise, which is a weakness, because FCM carries no details about spatial context which causes it to be sensitive to image artifacts and noise. In this paper, the PIABC-FCM algorithm uses the reformulated objective function as in [7]. In particular, they reformulated the objective function of FCMs based on the cluster center’s calculations only, while the matrix of membership U as in the original FCM objective function is unutilized. This consequently leads to a reduction in the sensitivity to noise.

The modified and reformulated objective function of the FCM is represented as follows.

(1) $$J_{i}=\sum_{j=1}^{n}\sum_{k\in C_{j}}h_{kj}^{2}*D_{kj}$$

Here, *h_kj_* is the value of membership which is different from the original membership value of FCM; it is employed to decrease noise sensitivity. The modified value of membership is computed as in Equation (2).

(2) $$h_{kj}=\mu_{\mathrm{kj}}-\frac{1-\mu_{k}{}_{j}}{2}$$

*Μkj* is represented by the pixel *k* belonging to the cluster of *j*th, and *Dkj* is the distance between the cluster center of the *j*th and *k*th pixel.

Indeed, to minimize the objective function, *Ji* represents the primary goal of reaching the best solution, which considers the optimal similarity through the pixels in one cluster.

## 4. The Original Artificial Bee Colony (ABC)

ABC is an optimization algorithm that is inspired by the searching nectar process of bees, developed by Karaboga and Basturk [46]. The population in the ABC algorithm is divided into three groups of bees. The first group comprises employed bees that search for sources of food and send information about food to the onlooker. The second group contains onlooker bees that perform more searches to select the best sources of food based on the fitness of food sources. After the employed bees abandon the sources of food, they become scout bees. The sources of food in the ABC algorithm are randomly initialized based on Equation (3)

(3) $$x_{i,j}=x_{\min,j}+rand(0,1)(x_{\max,j}-x_{\min,j})$$

*i* = 1, …, *SN*, the number of food sources is indicated as *SN*, *j* = 1, 2, …, *D*, the number of parameters are indicated as *D*, and values of *X_max,j_* and *x_min,j_* presented the upper and lower of dimension *j*, respectively. The new food source has been calculated in Equation (4).

(4) $$v_{ij}=x_{ij}+\varphi_{ij}(x_{ij}-x_{k.j})$$

j∈(1,2,…,D) is randomly chosen, where K∈(1,2,…,SN) and $K\neq i.\varphi_{ij}$. The source of food fitness value (solutions) *fit*(*x_i_*) has been calculated using *f*(*x_i_*) as in Equation (5).

(5) $$fit(x_{i})=\{\begin{array}{cc} \frac{1}{\left(1+f(x_{i})\right)} & iFf(x_{i})\geq 0\\ 1+abs(f(x_{i})) & iFf(x_{i})<0 \end{array}$$

The bee onlooker chooses a food source using a probability (*P*) which correlates with the fitness value participated by the employed bees. The value of *P* was determined based on Equation (6).

(6) $$P^{i}=\frac{fit_{i}}{\sum_{n=1}^{sn}fit_{i}}$$

When the solutions that cannot be enhanced have reached the “limit,” the employed convert to scout and abandon the solutions. Then, the new scout starts the new search space as expressed in Equation (4). These phases are executed until the stop criterion is reached.

## 5. Improved Global Best-Guided Artificial Bee Colony Algorithm (IABC) Based on a New Probability Model

### 5.1. IABC

In Cao et al. [47], a new variant of ABC-based global best guided called IABC was proposed. IABC was aimed at improving the performance of ABC based on global best guided (GABC) [48] by modifying the search equation in the onlooker bee phase to improve the exploitation capability. In GABC [48], the modification of the search equation is defined by the following:

(7) $$v_{ij}=x_{ij}+\varphi_{ij}(x_{ij}-x_{k.j})+\psi_{ij}\left({Gbest}_{j}-x_{i.j}\right)$$

where *Gbest* is the global best solution found so far and $\psi_{ij}$ is the random number between [0, *Z*], and *Z* is a positive constant number. In IABC, Cao, Lu, Pan and Sun [47] modified the onlooker search equation to improve the exploitation capability as follows:

(8) $$v{*}_{ij}=a_{1}.x_{ij}+a_{2}.{Gbest}_{j}+a_{3}.(x_{k.j}-x_{ij})$$

The weights *a*_1_, *a*_2_, and *a*_3_ are three random values, and *a*_1_ + *a*_2_ + *a*_3_ = 1 is formed by generating three random numbers *a*_1_, *a*_2_, and *a*_3_ in the range [0, 1]. Then, the Equations (9)–(11) are used to generate new weights:

(9) $$a_{1}=\frac{a_{1}}{a_{1}+a_{2}+a3_{3}}$$

(10) $$a_{2}=\frac{a_{2}}{a_{1}+a_{2}+a3_{3}}$$

(11) $$a1=\frac{a_{3}}{a_{1}+a_{2}+a3_{3}}$$

In IABC, Cao, Lu, Pan and Sun [47] used the parameter *MR* to improve the convergence speed as in [49] which is introduced to control the number of updating dimensions *j*; *MR* is used to update the search equation in the onlooker phase as follows:

(12) $$v_{ij}=\left\{ \begin{array}{l} v{*}_{ij},\;(Eq\;8)\;\;if\;\;\;\;rand_{j}\;\;\;<MR\\ v_{ij},\;\;\;(Eq\;7),\;\;\;\;\;otherwise \end{array}\right.$$

### 5.2. IABC-Based New Probability Model

Accordingly, this paper proposes a new probability model that makes sure that onlooker bees have more attractive solutions to search when the good solutions are varied, and the selected good solution probability is significantly larger than the ones with a bad solution. In general, the balancing between exploration and exploitation has been used to evaluate the performance of ABC. Therefore, the exploitation process of the best solution in the current case and other good solutions are considered the main issue. IABC was proposed in [47] and it functioned very well in the exploitation process but relatively underperformed in the process of exploration. Moreover, when there is no substantial difference between the qualities of fitness values for all food sources, similar probability values are attained according to Equation (6), but these values do not fully exploit the good solutions. Consequently, a newly proposed probability model would ensure that the onlooker bee has chosen good solutions, which could improve the ABC performance. To address this problem, the authors propose a new model of probability as in Equation (13),

(13) $$p(x_{i})=\frac{0.5}{(e^{r(Xbest_{i})/SN)})\sqrt{{}^{r(Xbest_{i})}}}$$

where *p*(*x_i_*) is the chosen probability of the *i*th source of food and *r*(*X_best,i_*) is the ranking of the best sources of foods in ascending based on the objective function values. The pseudo-code of the PIABC-FCM algorithm is represented in Algorithm 1.
**Algorithm 1**: PIABC ** Initialization**
1:  The centroids value is initialized by Equation (3)2:  *Cycle*=3:  **For** each *food source(i)*4:  *Counter(i)* = 05:  End
  **Repeat Employee phase**
6:  **FOR** each *employee bee*(i)7:  Generate new solutions *v_ij_* in the neighbourhood of *x_ij_*  Using “**Equation (7)**
8:  **If**
*fit(v_ij_) ≥ fit(x_ij_)*  Change *x_ij_* by *v_ij_*  *Counter(i)* = 09:  **Else**  *Counter(i)*= *Counter(i)* + 1  **End if**  **End for**  **Onlooker Phase**10: “Calculate probability *P(x_i_)* for the solutions    **by Equation (13).**11: **FOR** each *food source(i)*12: Select a solution depending on *P(x_i_*).13: Generate new solution *v_ij(o)_* in the neighbourhood of *x_ij(o)_*
   Using “Equation (12).    Evaluate new solution *v_ij(o)_*
   **If**
*fit(v_ij(o)_) ≥ fit(x_ij(o)_)*
14: Change *x_ij(o)_* by *v_ij(o)_*
15: *Counter(i)* = 0   **Else**   “*Counter(i)* = *Counter(i)* + 116: **End if**   **End for**   **Scouts phase**17: **FOR** each *food source(i)*
18: **If**
*Counter(i) > limit*19: Abandon the food source(*i*)20: Generate new solutions using Equation (3)21: Memorize the best solution achieved so far.22: ***Cycle = Cycle + 1***23: **Until** termination criteria are reached


## 6. The Proposed Approach (PIABC-FCM)

In this paper, the proposed methods of PIABC-FCM are a clustering algorithm that uses the capability of the proposed PIABC to determine the best outcomes that are used as the cluster centers for FCM; the steps for PIABC-FCM are described as follows.

### 6.1. Initialization

The proposed methods of IABC-FCM and PIABC-FCM begin by initializing the *Np* food sources. Each food source *Np* is represented by a set of cluster centers (centroids) found by the proposed PIABC. The centroid value between the maximum and minimum intensity of the grey image is to be segmented. The centroid value is initialized by the food source initialization as in Equation (14)

(14) $$\begin{array}{cc} C_{i}=C_{low}+rand(0,1)*(C_{high}-C_{low}); & i=1,2,\ldots ,Np \end{array}$$

where *i* denotes the food source and *C_i_* is the *i*th cluster center of a particular food source as in Equation (15).

*Np* = *C*_1_, *C*_2_, *C*_3_, *C*_4_, …, *C_n_* (15)

Moreover, to determine the best centroids, the fitness function *Ji* as in Equation (1) is used. The minimal value of *Ji* is considered the best solution to check the similarity between the pixels of the same cluster.

### 6.2. Employed Bees Phase

This phase comprises the use of the proposed PIABC algorithm to find the best clusters centroid (food source) within the neighborhood position of pixels. For each of the food sources, a neighbor of its present position is selected using Equation (7), where *j* = [1, 2, *…,C_n_*], *i* = [1, 2, …, *Np*], *x_j,i_* is a randomly chosen *j*th parameter of the *k*th individual and *i* is one of the *Np* food sources with *i ≠ k*. If any of *v_i_’*s parameters exceed its set limits, it should be changed to meet the right range. With the creation of a new competitive food source, *v_ij_*, the fitness value of the neighbor is calculated as in Equation (16). Say, *fit_j_*. If *fit_i_ > fit_j_*; then, update the food source with the neighbor source, otherwise, keep it as it is.

(16) $$fit_{i}=\frac{1}{1+J_{i}}\;\;\;\;\;\;\;J_{i}>0\;$$

### 6.3. Onlooker Bees Phase

The onlooker bee selects a food source based on a likelihood that is proportional to the fitness of the food source that was used by employed bees. The calculation of the probability model of the PIABC-FCM is used as the new probability model proposed by PIABC as in Equation (13). After a source of food is selected, as in the employed bees phase, a neighbor source *v*_i_ is evaluated by Equation (12), and its fitness value of the candidate solution is computed. Then, a process of greedy selection is performed between *v*_i_ and *x_i_*. Therefore, by recruiting more onlookers to richer sources, positive feedback behavior appears.

### 6.4. Scout Bees Phase

When the solutions that cannot be enhanced have reached the “limit,” the employed bees convert to scout bees and abandon the solutions. Then, the new bees of scout start the new search as expressed by Equation (14). These phases are executed until the stop criterion is reached.

### 6.5. Segmentation

The solution vector has the highest fitness level, hence it is chosen and used as the FCM’s initial cluster center values. The values of the cluster centers will alter in this scenario until the separation among pixels in the same cluster (inter-cluster) meets the minimal value. The pseudo-code and flowchart of the PIABC-FCM algorithm are represented in Algorithm 2.
**Algorithm 2:** PIABC-FCM  **Initialization**
1:  The centroids value is initialized by Equation (14)2:  *Cycle* = 13:  **For** each *food source(i)*4:  *Counter(i)* = 05:  End  **Repeat Employee phase**
6:  **FOR** each *employee bee*(i)7:  Generate new solutions *v_ij_* in the neighbourhood of *x_ij”_*  using **Equation (7)**
8:  Calculate the membership matrix *h_kj_* using Equation (2)  Calculate the fitness value fit for the new solution  by Equation (1) and Equation (16) 9:  **If **
*fit(v_ij_) ≥ fit(x_ij_)*10: Change *x_ij_* by *v_ij_*11: *Counter(i)* = 012: **Else**
13: *Counter(i)* = *Counter(i)* + 114: **End if**15: **End for**   **Onlooker Phase**16: Calculate probability *P(x_i_*) for the solutions   **by Equation (13).**17: **FOR** each *food source(i)*18: Select a solution depending on *P(x_i_*).19: “Generate new solution *v_ij(o)_* in the neighbourhood of *x_ij(o)_*   Using **Equation (12)**.    Evaluate new solution *v_ij(o)_*   Calculate the membership matrix *h_kj_* using Equation (2)20: Calculate the fitness value fit for the new solution21: by Equation (1)and Equation (16) 22: **If**
*fit(v_ij(o)_) ≥ fit(x_ij(o)_)*23: Change *x_ij(o)_* by *v_ij(o)_*24: *Counter(i)* = 025: **Else**26: *Counter(i)* = *Counter(i)*+ 127: **End if**28: **End for**29: **Scouts phase**30: **FOR** each *food source(i)*31: **If**
*Counter(i) > limit*32: Abandon the food source(*i*) 33: Generate new solutions using Equation (14)34: Memorize the best solution achieved so far.35: ***Cycle = Cycle +***36: **Until** termination criteria are reached37: “Do the segmentation image by    “The optimal cluster centres for FCM.


## 7. Experiments and Results

The proposed method of the PIABC-FCM fuzzy clustering algorithm is conducted as a semi-automatic grey scale image segmentation algorithm to segment the given image with a known number of clusters (regions), while the images do not care about the number of clusters. This section is divided into two parts where the first part contains cluster validity indices that are used to evaluate solutions of cluster algorithm. Meanwhile, to evaluate the quality of the PIABC-FCM clustering algorithm, the second part presents the experiments conducted on natural images.

### 7.1. Cluster Validity Indices

The cluster validity indices are used to evaluate the solutions which come from clustering algorithms with different algorithms and settings. In other words, to reach a better fuzzy clustering solution between a set of candidate solutions, an efficient index is required to determine the quality of the fuzzy partitions’ outcomes. These indices are based on two main criteria, namely, compactness and separation.

This section discusses the validity indices that evaluate the clustering approaches. Moreover, the quality of the PIABC-FCM fuzzy clustering algorithm is evaluated by the partition coefficient PC [9], classification entropy (CE) [9,50], SC [51], and separation index (S) and partition coefficient (PC) index as a well-known fuzzy cluster validity measurement [9]. This index uses values of membership (compactness) in their calculations, and thus, the advantage of this index is that it is easy to evaluate. However, to obtain proper clustering outcomes,PC∈[1/c,1] the index must be maximized. The index is defined as in Equation (17).

(17) $$Vpc=\frac{1}{n}\sum_{j=1}^{c}\sum_{i=1}^{n}u_{ij}^{2}$$

The classification entropy (CE) function [50] is defined in Equation (18)

(18) $$CE=-\frac{\sum_{j=1}^{N}\sum_{i=1}^{c}u_{ij}*log\;u_{ij}}{N}$$

where *N* represents the number of pixels and c represents the number of clusters, and *u_ij_* is the membership value of the *i*th data point to the *j*th cluster. The minimum value of CE shows the best classification, and the disadvantages of CE are the same as those of PC; they measure only the fuzzy partition and there is a lack of the use of the property of the data themselves.

The index of the subarea coefficient (SC) [51], which computes the ratio of the sum of separation and compactness of the clusters, is shown in Equation (19).

(19) $$\mathrm{SC}=\sum_{i=1}^{c}\frac{\sum_{k=1}^{n}{\left(u_{ik}\right)}^{m}{\|x_{k}-v_{i}\|}^{2}}{n_{i}\sum_{j=1}^{c}\;\|v_{j}-{v_{i}\|}^{2}}$$

The index of separation (S) [3], which calculates the partition validity of a minimum-distance separation, is shown in Equation (20) below:

(20) $$S=\frac{\sum_{i=1}^{c}\sum_{k=1}^{n}{\left(u_{ik}\right)}^{2}{\left\|x_{k}-v_{i}\right\|}^{2}}{n_{1\leq i\leq c,\;1\leq k\leq n}\;{\left\|x_{j}-v_{i}\right\|}^{2}}$$

### 7.2. Natural Images

The experiments were conducted using benchmarked natural images obtained from the Berkeley1 segmentation dataset [3,35,52]. These images were grey-scale images in JPEG format such as Lena, baboon, pepper, airplane, and cameraman with varying levels of complexity. All natural image sizes are 512 × 512 except the pepper image which is 256 × 256. In this experiment, the PIABC-FCM outcomes were compared with other related works such as MoABC-FCM, PSO-FCM, ABC-FCM, MABC-FCM, and FCM, which were reported in a study of Handling Fuzzy Image Clustering with a Modified ABC algorithm [35]. Moreover, the outcomes were compared with recent related works, especially those on hybrid biogeography-based fuzzy C-means such as BBO-FCM and EBO-FCM [3].

To obtain the best outcomes from any optimization algorithm, a suitable selection of parameters is very critical because these parameters are important in the algorithm’s performance and accuracy. The values of parameters of PIABC-FCM are population size *SN* (*Np*), *limit*, maximum cycle number (*MCN*), nonnegative constant parameter *Z*, and the weighted exponent *m*. The values of these parameters are set based on other state-of-the-art methods [35,53]. Table 1 presents the summary of parameter values from the state-of-the-art methods. Moreover, the parameter settings of hybrid biogeography-based fuzzy C-means algorithms are available in related works [3].

In Table 2, the validity indices of fuzzy clustering algorithms such as FCM, PSO-FCM, ABC-FCM, MABC-FCM, MoABC-FCM, AFSA–FCM, BBO–FCM, and PIABC-FCM are evaluated using cluster validity indices, classification entropy (CE), partition coefficient (PC), subarea coefficient (SC), and separation index (S), where the minimum value for CE and SC represents the best value, and the maximum value for PC and S represents the best value. Table 2 provides the validity indice outcomes of all clustering approaches for the Lena image with clusters number equal to five, and the bold items represent the better outcomes obtained by the clustering approaches for the Lena image.

**Figure 1:**
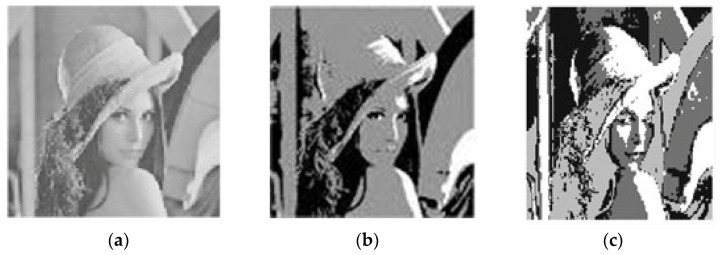
Lena segmented the image by (**a**) original image, (**b**) PIABC-FCM, and (**c**) IABC-FCM.

To further help the process of evaluation, the results in Table 2 were classified into two groups and bold font indicates best result obtained. The first group contains CE, SC, and PC validity indices of clustering. The table clearly shows that PIABC-FCM outcomes are better than the other related works of clustering algorithms; the PIABC-FCM yields better results because PIABC has a good ability in balancing between exploration and exploitation. Moreover, PIABC has a good ability in exploring search space (image pixels), which leads to the effective exploration of the whole region. The second group contains S validity indices, and it is clear that PIABC-FCM outperforms all of the clustering approaches and the rest of the PSO-FCM in the S index, respectively. Accordingly, Figure 1 represents Lena’s original image and segmented image by PIABC-FCM and IABC-FCM.

**Figure 2:**
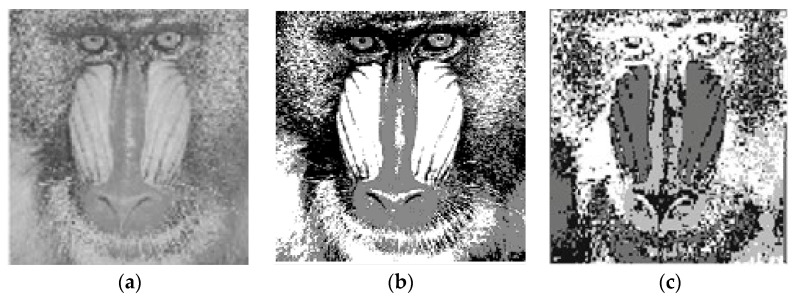
Baboon segmented image: (**a**) original image, (**b**) PIABC-FCM, and (**c**) IABC-FCM.

**Figure 3:**
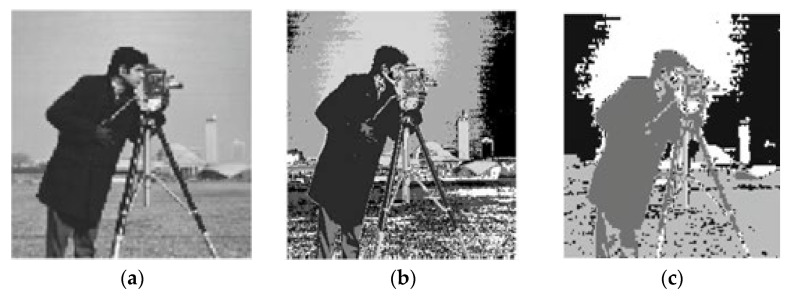
Cameraman segmented image: (**a**) original image, (**b**) PIABC-FCM, and (**c**) IABC-FCM.

In Table 3 and Table 4, the validity indice outcomes of all clustering approaches for the baboon and cameraman images with cluster numbers equal four and five and bold font indicates best result obtained. In this regard, the tables are showing the superiority of PIABC-FCM to IABC-FCM and other clustering approaches in cases of CE, SC, and PC validity indices. Here, the EBO–FCM and PSO-FCM are better than PIABC-FCM and other clustering approaches in the S validity index for the baboon and cameraman images because S measures the separation (dissimilarity) between clusters (intra cluster distance). The maximum value of S presents the best separation between clusters and it is important when there is an unknown appropriate number of clusters in the image dataset. However, in these experiments, the numbers of clusters in the baboon and cameraman images are known, which are four and five. Figure 2 presents the original Baboon image and segmented image by PIABC-FCM and IABC-FCM. Moreover, Figure 3 presents the original cameraman image and segmented image by PIABC-FCM and IABC-FCM algorithms.

**Figure 4:**
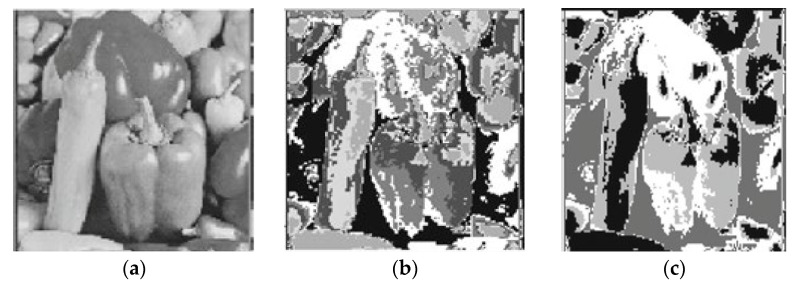
Pepper segmented image: (**a**) original image, (**b**) PIABC-FCM, and (**c**) IABC-FCM.

In Table 5, the validity indices outcomes of all clustering approaches for the pepper image with clusters number equal to five and bold font indicates best result obtained, and it can be clearly shown that PIABC-FCM outcomes are better than IABC-FCM and other clustering algorithms. Thus, the capability of PIABC is improved in finding a globally optimal solution (best solutions) based on its capability to find a good balance between the exploitation of found-so-far positions and the exploration of the search space. On the other hand, other methods are used in conventional optimization algorithms. Relevantly, FCM suffered from many weaknesses such as being stuck in local optima, the inability to explore the whole search space because they can search in one direction only, and the use of conventional FCM which suffered from sensitivity to noise problems. Figure 4 presents the original pepper image and the segmented image by PIABC-FCM and IABC-FCM algorithms.

**Figure 5:**
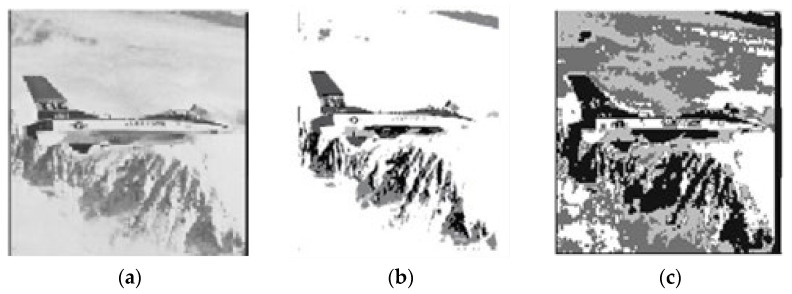
Airplane segmented image: (**a**) original image, (**b**) PIABC-FCM, and (**c**) IABC-FCM.

In Table 6, the validity indices outcomes of all clustering approaches for the airplane image with clusters number equal to five are shown and bold font indicates best result obtained. As seen in the table, PIABC-FCM outperforms all clustering approaches, while PSO-FCM is better than other related algorithms and is close to PIABC-FCM in separation index S which presents the best separation between clusters when the number of clusters is unknown in certain datasets. Figure 5 accordingly presents the original airplane image and segmented image by PIABC-FCM and IABC-FCM algorithms.

PIABC-FCM has proven the capability to reach the optimal initialize cluster centers (centroid location) and avoid being stuck in local optimum when compared with IABC-FCM and other related works [3,35], based on the similarity between pixels at the same cluster (inter-cluster). In this paper, PIABC-FCM and IABC-FCM obtained the semi-automatic segmentation and this is suitable when the type of image dataset is unrequested and the numbers of clusters are known by experts.

**Figure 6:**
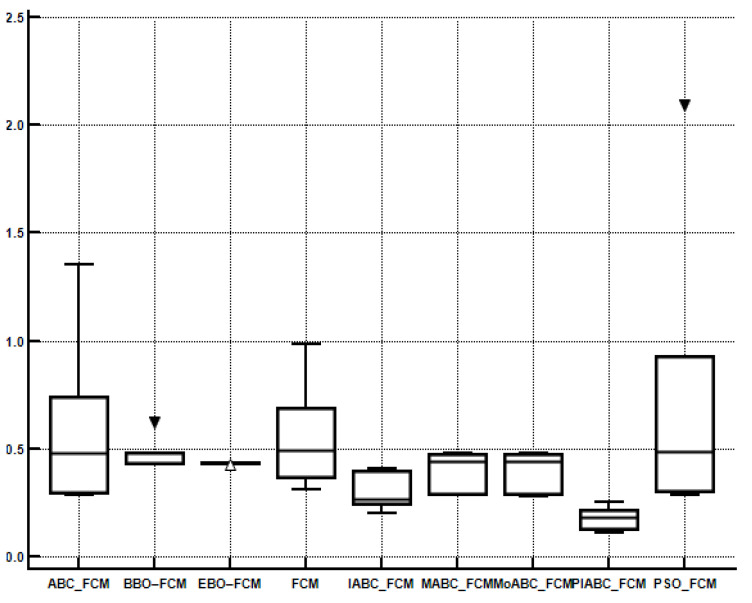
Box-whisker graph to present the outcomes of CE index.

**Figure 7:**
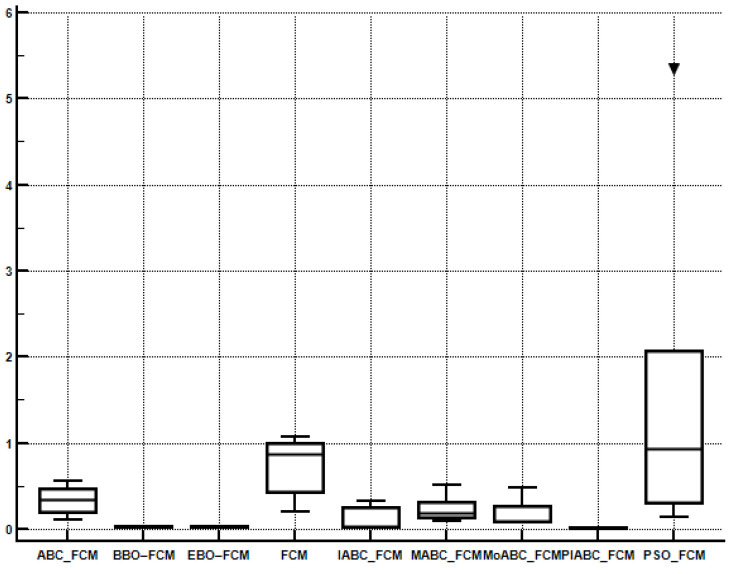
Box-whisker graph to present the outcomes of the SC index.

**Figure 8:**
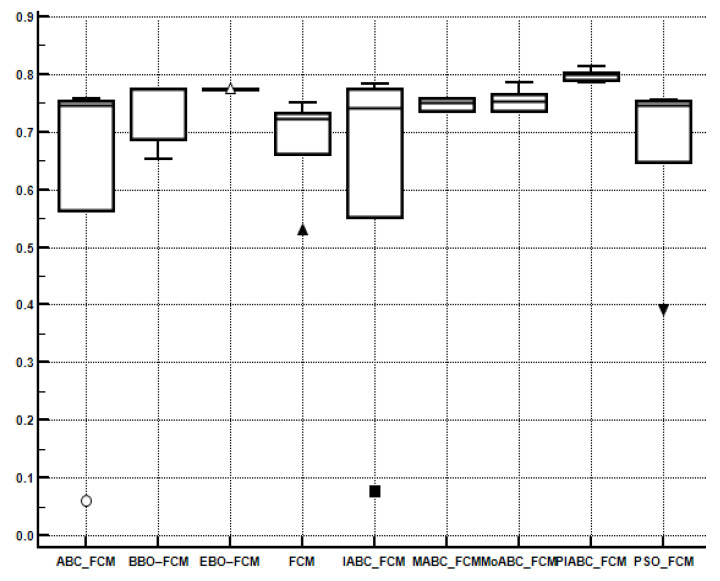
Box-whisker graph to present the outcomes of the PC index.

**Figure 9:**
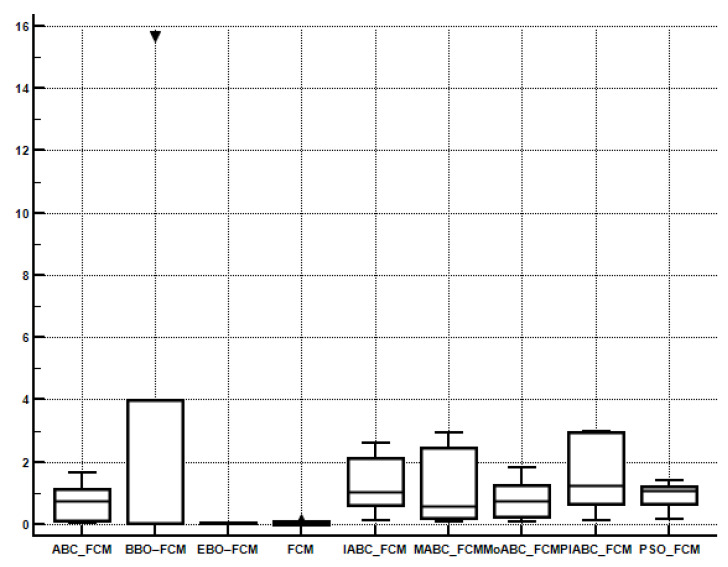
Box-whisker graph to present the outcomes of the S index.

In order to compare the performance of multiple algorithms on the test suite, the Friedman test is obtained. Table 7, Table 8, Table 9 and Table 10 present the average rankings of the proposed algorithm. PIABC_FCM outperforms IABC-FCM and other related algorithms such as BBO–FCM, MoABC_FCM, EBO–FCM, MABC_FCM, ABC_FCM, PSO_FCM, and FCM. In Table 7 and Table 8, the best value is the minimum value because the minimum values of validity indices of CE and SC are considered the best values. Meanwhile, in Table 9 and Table 10, the best value is the maximum value because the maximum values of validity indices PC and S are the best values as shown in bold. As seen, the performance of the above clustering algorithms is sorted by average ranking into the following order: The best average ranking was obtained by the PIABC_FCM, which outperforms the others. Moreover, in Figure 6, Figure 7, Figure 8 and Figure 9, the Box-whisker graphs present all outcomes of five neutral images based on clustering validity indices CE, SC, PC, and S.

## 8. Conclusions

In this paper, an unsupervised clustering algorithm based on a new search probability model based on an improved global best-guided artificial bee colony algorithm (IABC), namely, PIABC, was proposed to improve the balance between exploration and exploitation of IABC. Finally, an unsupervised clustering-algorithm-based (PIABC) model called (PIABC-FCM) was proposed for the selection of centroid locations. The new search probability model (PIABC) can improve exploration by choosing the best source of food and directly affects the exploitation process in IABC and the selection of centroid locations in FCM. The experiments and results were obtained from natural images. The use of PIABC-FCM shows promising results when compared with IABC-FCM and other comparable methods.

## Figures and Tables

**Table 1:** The Summary of Parameters Values from state-of-the-art methods.

Approach	Population Size *SN* (*Np*)	*Limit*	Maximum Cycle Number (*MCN*)	Nonnegative Constant Parameter *Z*	Weighting Exponent *m*
PIABC-FCM	100	100	2000	1.43	2
ABC-FCM [35]	100	100	2000	1.43	2
MABC-FCM [35]	100	100	2000	1.43	2
MoABC-FCM [35]	100	100	2000	1.43	2
ABC-SFCM [53]	20	100	2000	NA	2

**Table 2:** Outcomes of PIABC-FCM and other clustering algorithms on Lena image.

Images	Algorithms	CE	SC	PC	S
LENA C = 5	FCM	0.9850	1.0684	0.5316	1.0742 × 10^−4^
	PSO-FCM	2.0900	5.3410	0.3925	**1.4035**
	ABC-FCM	1.3544	0.5670	0.0608	0.1458
	MABC-FCM	0.4714	0.5129	0.7364	0.5962
	MoABC-FCM	0.4712	0.4840	0.7364	0.7261
	EBO–FCM	0.4335	0.0273	0.7745	0.0591
	BBO–FCM	0.4335	0.0266	0.7745	0.059
	IABC-FCM	0.2619	0.0348	0.7125	1.025
	PIABC-FCM	**0.1128**	**0.0215**	**0.8144**	1.2370

**Table 3:** Outcomes of PIABC-FCM and other clustering algorithms on the baboon image.

Images	Algorithms	CE	SC	PC	S
Baboon C = 4	FCM	0.5897	0.8642	0.7063	0.1862
	PSO-FCM	0.5372	0.9271	0.7509	1.0964
	ABC-FCM	0.5316	0.3460	0.7510	1.6614
	MABC-FCM	0.4388	0.1522	0.7511	2.9273
	MoABC-FCM	0.4372	0.1003	0.7524	1.8163
	BBO–FCM	0.4294	0.0260	0.7763	0.0569
	EBO–FCM	0.6186	0.0355	0.6532	**15.6874**
	IABC-FCM	0.3903	0.0370	0.7721	2.6155
	PIABC-FCM	**0.1790**	**0.0201**	**0.7917**	2.9803

**Table 4:** Outcomes of PIABC-FCM and other clustering algorithms on the Cameraman image.

Images	Algorithms	CE	SC	PC	S
Cameraman C = 5	FCM	0.3104	0.1960	0.7515	0.0031
	PSO-FCM	0.3086	0.1441	0.7564	**1.1370**
	ABC-FCM	0.3052	0.1108	0.7574	0.7516
	MABC-FCM	0.2968	0.0995	0.7583	0.2269
	MoABC-FCM	0.2964	0.0931	0.7868	0.3214
	BBO–FCM	0.4326	0.0264	0.7748	0.0598
	EBO–FCM	0.4326	0.0261	0.7745	0.0571
	IABC-FCM	0.2670	0.3194	0.0772	0.7924
	PIABC-FCM	**0.1981**	**0.0202**	**0.7990**	0.8690

**Table 5:** Outcomes of PIABC-FCM and other clustering algorithms on the pepper image.

Images	Algorithms	CE	SC	PC	S
Pepper C = 5	FCM	0.4896	0.9661	0.7249	0.0262
	PSO-FCM	0.4852	0.9725	0.7326	0.8170
	ABC-FCM	0.4824	0.4251	0.7330	0.9293
	MABC-FCM	0.4810	0.2537	0.7343	2.2715
	MoABC-FCM	0.4810	0.1981	0.7344	1.0421
	BBO–FCM	0.4324	0.0262	0.7748	0.0569
	EBO–FCM	0.4326	0.0349	0.6991	0.0571
	IABC-FCM	0.4051	0.2273	0.7410	1.9552
	PIABC-FCM	**0.2539**	**0.0182**	**0.7869**	**2.9160**

**Table 6:** Outcomes of PIABC-FCM and other clustering algorithms on airplane image.

Images	Algorithms	CE	SC	PC	S
Airplane C = 5	FCM	0.3848	0.5172	0.7237	0.0016
	PSO-FCM	0.2858	0.3640	0.7466	**0.1520**
	ABC-FCM	0.2831	0.2257	0.7470	0.0317
	MABC-FCM	0.2825	0.1868	0.7567	0.0954
	MoABC-FCM	0.2810	0.0935	0.7567	0.0982
	BBO–FCM	0.4323	0.0258	0.7748	0.0576
	EBO–FCM	0.4323	0.0258	0.7748	0.0578
	IABC-FCM	0.2004	0.0164	0.7841	0.1123
	PIABC-FCM	**0.1308**	**0.0115**	**0.7990**	0.1164

**Table 7:** Friedman Test based on CE validity index—result for natural images.

Algorithm	Mean Rank
PIABC_FCM	1
IABC_FCM	2
MoABC_FCM	4.1
MABC_FCM	4.9
EBO–FCM	5.3
ABC_FCM	6.2
BBO–FCM	6.7
PSO_FCM	7.2
FCM	7.6

**Table 8:** Friedman Test based on SC validity index—result for natural images.

Algorithm	Mean Rank
PIABC_FCM	1
BBO–FCM	2.7
EBO–FCM	2.7
MoABC_FCM	4.6
IABC_FCM	4.8
MABC_FCM	5.8
ABC_FCM	6.8
FCM	8.2
PSO_FCM	8.4

**Table 9:** Friedman Test based on PC validity index—result for natural images.

Algorithm	Mean Rank
PIABC_FCM	9
EBO–FCM	7.4
MoABC_FCM	6
IABC_FCM	5.4
MABC_FCM	5
BBO–FCM	4.4
ABC_FCM	3.2
PSO_FCM	2.6
FCM	2

**Table 10:** Friedman Test based on S validity index—result for natural images.

Algorithm	Mean Rank
PIABC_FCM	8.2
IABC_FCM	6.8
PSO_FCM	6.8
MABC_FCM	5.8
MoABC_FCM	5.6
ABC_FCM	4.2
BBO–FCM	4
EBO–FCM	2.4
FCM	1.2

## Data Availability

https://www2.eecs.berkeley.edu/Research/Projects/CS/vision/bsds/.
